# Hepatic Adverse Effects of Fructose Consumption Independent of Overweight/Obesity

**DOI:** 10.3390/ijms141121873

**Published:** 2013-11-05

**Authors:** Alini Schultz, Debora Neil, Marcia B. Aguila, Carlos A. Mandarim-de-Lacerda

**Affiliations:** Laboratory of Morphometry, Metabolism and Cardiovascular Disease, Biomedical Center, Institute of Biology, State University of Rio de Janeiro, Av. 28 de Setembro 87 fds, Rio de Janeiro 20551-030, Brazil; E-Mail: schultz.alini@gmail.com (A.S.); deborasneilm@gmail.com (D.N.); marciaguila@gmail.com (M.B.A.)

**Keywords:** high fructose diet, high fat diet, insulin resistance, nonalcoholic fatty liver disease, nonalcoholic steatohepatitis

## Abstract

The chronic intake of fructose has been linked to insulin resistance, obesity, dyslipidemia and nonalcoholic fatty liver disease (NAFLD), which in turn, may progress to nonalcoholic steatohepatitis (NASH). We aimed to evaluate the magnitude of the effects of the chronic consumption of high-fructose (HFr) and high fat (HF) alone or combined. Four groups of male mice were fed different diets for 16 weeks: standard chow (9% fat: SC), HF diet (42% fat), HFr diet (34% fructose) and HF/HFr diet (42% fat, 34% fructose). The food intake was not different among the groups, and the body mass was not greater in the HFr group than in the SC group. The homeostasis model assessment for insulin resistance (HOMA-IR), as well as plasmatic total cholesterol and triglycerides were greater in the groups HF, HFr, and HF/HFr group than in the SC group. We observed in the groups HF, HFr and HF/HFr, compared to the group SC, nonalcoholic fatty liver disease (NAFLD) with a predominance of lipogenesis mediated by SREBP-1c and PPAR-γ, and a reduction of the oxidation mediated by PPAR-α. We also observed an increase in gluconeogenesis mediated by the GLUT-2 and the PEPCK. Importantly, we identified areas of necroinflammation indicating a transition from NAFLD to nonalcoholic steatohepatitis in the HFr and HF/HFr groups. This study is relevant in demonstrating that fructose consumption, even in the absence of obesity, causes serious and deleterious changes in the liver with the presence of the dyslipidemia, insulin resistance (IR), and NAFLD with areas of necroinflammation. These conditions are associated with a poor prognosis.

## 1. Introduction

Fructose is a monosaccharide found in fruits and ingested as a sweetener in processed foods, soda, and high fructose corn syrup, the latter of which is commonly used in juices, breakfast cereals, and prepackaged foods [1]. Moreover, there is growing evidence suggesting that, due to the peculiar characteristics and harmful effects on metabolism, chronic ingestion of fructose can also induce each of the phenomena associated with the metabolic syndrome [2,3].

Globally, countries with a higher availability of high-fructose corn syrup have a higher prevalence of type 2 diabetes, independent of obesity [4]. Concomitant with the increased presence of fructose in the diet increases the incidence of obesity, insulin resistance (IR), and systemic arterial hypertension [4,5].

Increased calorie intake, especially refined sugar and fructose, correlates with increases in dyslipidemia, IR, and nonalcoholic fatty liver disease (NAFLD) [3,6]. In addition, NAFLD is a clinical manifestation of IR in the liver commonly seen with the chronic intake of a high-fat diet [7,8]. NAFLD is the first step in the hepatic diseases that can evolve into steatohepatitis (NASH), with inflammatory infiltration resulting in cirrhosis and even hepatocarcinomas [9]. The recent literature has convincing evidence indicating that the continuous consumption of fructose can lead to NAFLD and NASH in humans [2,10].

This study was undertaken to compare the impact of the effects of two different types of dietary regimens, one with high-fructose content and the other with high-fat content, as well as the combination of the two regimens, and also on alterations in lipid and carbohydrate metabolism, adipose tissue, the endocrine pancreas and the liver.

## 2. Results and Discussion

### 2.1. Body Mass and Intake of Food

**Figure 1:**
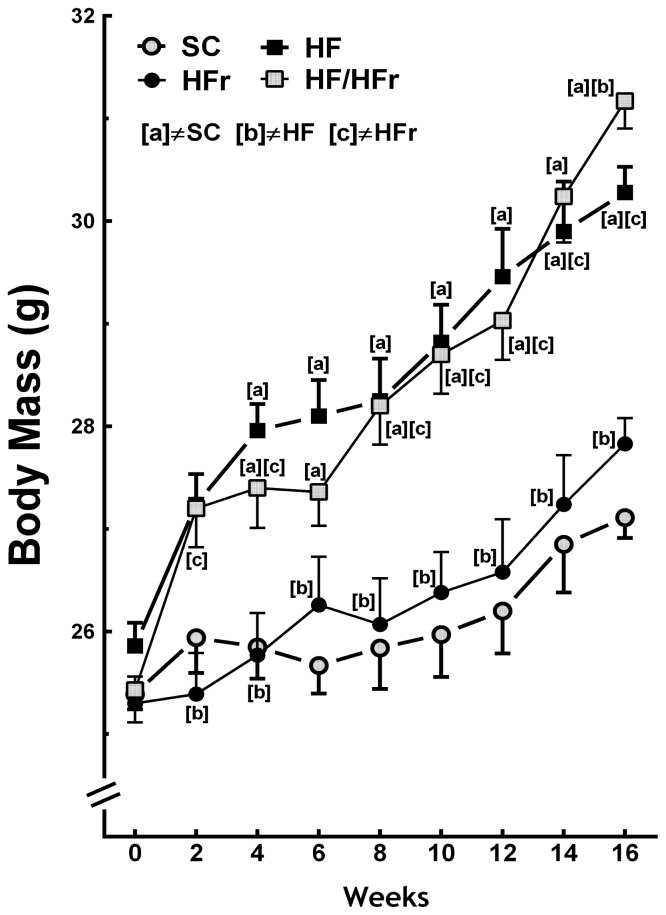
Body mass. Diets were administered for 16 weeks. SC (standard chow group), HF (high-fat group), HFr (high-fructose group), HF/HFr (high-fat and high-fructose group). Values are means ± standard error. There was a significant difference (*p* ≤ 0.05 for the same week) compared with the ^a^ SC group; ^b^ HF group; and ^c^ HFr group.

At the end the experiment the BM (body mass) of the HF and the HF/HFr group animals were 12% (*p* < 0.001) and 15% (*p* < 0.001) greater, respectively, than the BM of the SC (standard chow) group animals. The BM of the HF/HFr group was 12% greater than that of the HFr group (*p* < 0.001). However, the BM of the HFr group was smaller than that of the SC group (Figure 1). In addition, the food intake (g/animal/week) was not significantly different among the groups studied (Table 1). The energy intake (kcal/animal/week) corresponded with the results of the BM measurements. No significant differences were found between the SC group and the HFr group or between the HF group and the HF/HFr group. Because of the higher energy density of the diets containing high levels of fat, energy intake was 11% higher in the HF group (*p* < 0.001) and 10% higher in the HF/HFr group (*p* < 0.001) than in the SC group. The energy intake was 12% higher in the HF/HFr group than in the HFr group (*p* < 0.001) (Table 1).

The present study demonstrated that fructose consumption did not lead to increases in BM compared with consumption of the control diet. The changes in BM were observed only in the groups that consumed the high-fat diets alone or combined. However, animals that consumed fructose showed changes in metabolic and liver parameters similar to animals fed high fat diet. Demonstrating that consumption of a diet rich in fructose long term, even at a low percentage, is deleterious as fat. Remember that SC and HFR diets were isocaloric and therefore had no impact on the BM. However, the other results in the liver were due to the type of carbohydrate, fructose, and lipid alone or in combination. It is important to note that the increasing use of fructose as a sweetener contributes an average of greater than 400 kcal/day/person in the world, which correlates with the epidemiological data for increases in obesity. However, the effects of fructose seem, in part, to be independent of increases in BM [5,11].

### 2.2. OGTT (Oral Glucose Tolerance Test), IPITT (Intraperitoneal Insulin Tolerance Test) and Insulin

We compared the results of these tests for the animals fed the modified diets with those of the SC group. The area under the curve (AUC) for OGTT (oral glucose tolerance test) was higher in the HF group (+15%; *p* < 0.05), HFr group (+16%; *p* < 0.01), and HF/HFr group (+12%; *p* < 0.05) than in the SC group (Table 1). Similarly, the AUC for IPITT was higher in the HF group (+36%, *p* < 0.05), HFr group (+49%; *p* < 0.01), and HF/HFr group (+38%, *p* < 0.05) than in the SC group. Plasma insulin concentration was also studied and compared to the SC group. The plasma insulin concentration was higher in the HF group (+68%; *p* < 0.001), HFr group (+49%; *p* < 0.01), and HF/HFr group (+47%; *p* < 0.01) than in the SC group. Consequently, the HOMA-IR (homeostasis model assessment for insulin resistance) was greater in the HF group (+295%; *p* < 0.001), HFr group (+194%; *p* < 0.001), and HF/HFr group (+194%; *p* < 0.01) than in the SC group (Table 1).

Fructose is a simple carbohydrate with a low glycemic index. However, it appears that medium and long-term fructose consumption is able to disrupt the insulin-signaling pathway, leading to a pattern of hyperglycemia accompanied by compensatory hyperinsulinemia [12]. The hyperglycemia and hyperinsulinemia possibly occur because, in the liver, fructose activates the mitogen-activated protein kinases (MAPK) MKK7 and MAPK8, inducing phosphorylation of the insulin receptor IRS-1 in serine. This phenomenon in turn suppresses glucose uptake, leading to increased blood glucose levels and the concomitant increase in insulin secretion [2]. Although we did not evaluate the IRS-1, its phosphorylation reduction in the liver of the fructose-fed animals may be related to insulin resistance in this model [13]. Our data indicate that, in the context of carbohydrate metabolism, the consumption of fructose or lipids favored the development of insulin resistance with an increase of the HOMA-IR, plasma glucose and insulin.

### 2.3. Plasma Total Cholesterol, Triglycerides and Glucose

The total cholesterol was 49% higher in the HF group (*p* < 0.01), 37% higher in the HFr group (*p* < 0.05), and 68% higher in the HF/HFr group (*p* < 0.001) than in the SC group. Similarly, triglycerides were higher in all modified-diet groups compared to the SC group. These levels were 14% higher in the HF group (*p* < 0.001), 15% higher in the HFr group (*p* < 0.001), and 14% higher in the HF/HFr group (*p* < 0.001) than in the SC group. At the end of experiment, plasma glucose was 142% higher in the HF group (*p* < 0.001), 96% higher in the HFr group (*p* < 0.01), and 101% higher in the HF/HFr group (*p* < 0.01) relative to the SC group (Table 1).

### 2.4. Liver

The concentration of ALT was higher in the HF group (+134%; *p* < 0.001), HFr group (+115%; *p* < 0.01), and HF/HFr group (+102%; *p* < 0.01) than in the SC group (Table 1).

The results of the hepatic triglyceride analysis were similar to those of the plasma triglyceride analysis: the hepatic triglyceride levels were higher in the HF group (+69%; *p* < 0.001), HFr group (+24%; *p* < 0.05), and HF/HFr group (+48%; *p* < 0.001) than in the SC group. The triglyceride levels in the HF/HFr group were also significantly different from those in the HFr group (+19%; *p* < 0.05) (Table 1).

**Figure 2:**
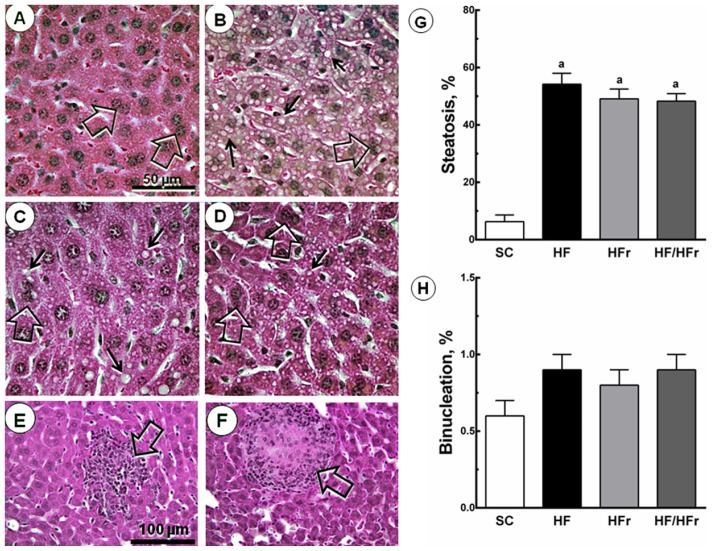
Volume density of hepatic steatosis and frequency of binucleation. The livers of the HF, HFr and HF/HFr groups showed increased NAFLD, steatotic macro- and microvesicles (**arrows**), and binucleation (**open arrows**). (**A**) SC; (**B**) HF; (**C**) HFr; and (**D**) HF/HFr (same magnification in **A** to **D**). Below, scattered necroinflammatory foci (**open arrows**) (**E**) in HFr group; and (**F**) in HF/HFr group (same magnification in **E** and **F**); The quantitative measure of (**G**) steatosis and (**H**) binucleation is shown in these graphs. Values are means ± standard error. There was a significant difference (*p* ≤ 0.05) compared with the ^a^ SC group.

There were also increases in hepatic steatosis in the HF group (+760%; *p* < 0.001), HFr group (+679%; *p* < 0.001), and HF/HFr group (+667%; *p* < 0.001) relative to the SC group. We also studied hepatic binucleation. Hepatic binucleation was observed at higher frequencies in the HF group (+50%; *p* < 0.001), HFr group (+33%; *p* < 0.01), and HF/HFr group (+50%; *p* < 0.001) than in the SC group (Figure 2). In addition, necroinflammatory foci were observed only in the HFr and HF/HFr groups (Figure 2).

**Figure 3:**
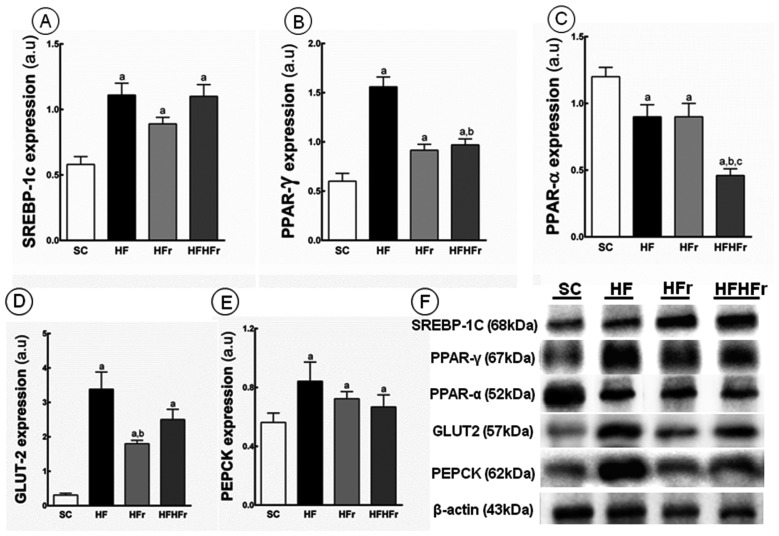
(**A**) Sterol regulatory element-binding proteins(SREBP)-1c; (**B**) peroxisome proliferator-activated receptors (PPAR)-γ; (**C**) PPAR-α; (**D**) glucose transporter (GLUT)-2; and (**E**) phosphoenolpyruvate carboxykinase (PEPCK) protein expression in hepatic tissue normalized to the signal for β-actin (expressed in arbitrary units, a.u.); (**F**) representative bands of the proteins. Values are means ± standard error. There was a significant difference (*p* ≤ 0.05) compared with the ^a^ SC group; ^b^ HF group; and ^c^ HFr group.

Compared to the SC group, increases in the hepatic expression of SREBP-1c were observed in the HF group (+91%; *p* < 0.001), HFr group (+53%; *p* < 0.05), and HF/HFr group (+90%; *p* < 0.001) (Figure 3A). Increased in the expression of PPAR-γ were observed in the HF group (+160%; *p* < 0.001), HFr group (+52%; *p* < 0.05), and HF/HFr (+62%; *p* < 0.01) (Figure 3B). In contrast, the hepatic expression of PPAR-α was lower in the HF group (−25%; *p* < 0.05), HFr group (−25%; *p* < 0.05), and HF/HFr group (−62%, *p* < 0.001) than in the SC group. The group HF/HFr was the most affected with lower hepatic expression of PPAR-α in comparison to the HF group (−49%, *p* < 0.01), and to the HFr group (−49%, *p* < 0.01) (Figure 3C).

Compared to the SC group, we observed an increase in PEPCK in the HF group (+113%; *p* < 0.001), in the HFr group (+68%; *p* < 0.05), and in the HF/HFr group (+74%; *p* < 0.01) (Figure 3E). In addition, still compared to the SC group, the GLUT2 expression was increased in the HF group (+990%; *p* < 0.001), in the HFr group (+481%; *p* < 0.01), and in the HF/HFr group (+706%; *p* < 0.001) (Figure 3D).

**Figure 4:**
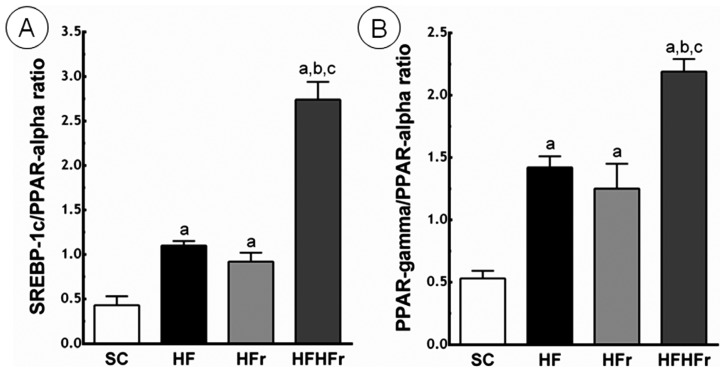
(**A**) Sterol regulatory element-binding proteins (SREBP)-1c/peroxisome proliferator-activated receptors (PPAR)-α ratio; (**B**) PPAR-γ/PPAR-α ratio. Expressed in arbitrary units, a.u. SC, HF, HFr and HF/HFr. Values are means ± standard error. There was a significant difference (*p* ≤ 0.05) compared with the ^a^ SC group; ^b^ HF group; and ^c^ HFr group.

Compared to the SC group, the ratio between SREBP-1c and PPAR-α was greater in the HF group (+156%; *p* < 0.01), in the HFr group (+114%; *p* < 0.05), and in the HF/HFr group (+537%; *p* < 0.001). The HF/HFr group showed the greatest the ratio between SREBP-1c and PPAR-α, greater than the HF group (+149%; *p* < 0.001), and greater than the HF/HFr group (+198%; *p* < 0.001) (Figure 4).

Compared to the SC group, the ratio between PPAR-γ and PPAR-α was greater in the HF group (+168%; *p* < 0.001), in the HFr group (+136%; *p* < 0.01), and in the HF/HFr group (+313%; *p* < 0.001). Again, the group HF/HFr showed the greatest values of the ratio between PPAR-γ and PPAR-α, greater than the HF group (+54%; *p* < 0.001), and the HFr group (+75%; *p* < 0.001) (Figure 4).

In the liver, fructose directly and indirectly inhibits the oxidation of free fatty acids (FFA) [14]. In the direct pathway, the continuous production of acetyl-CoA because of the metabolism of fructose exceeds the mitochondrial capacity for its metabolism (Krebs cycle), and acetyl-CoA is thus converted to citrate. Citrate is the fuel for the process known as *de novo lipogenesis* (*DNL*), which contributes to increased hepatic lipogenesis. Acetyl-CoA is subsequently converted to malonyl-CoA, which inhibits the activity of carnitine palmitoyl transferase (CPT-1), thus preventing the mitochondrial oxidation of FFA [15]. In addition, fructose activates transcription factors, including SREBP-1c, and ChREBP (carbohydrate response element binding protein), which in turn, control the synthesis of *DNL* enzymes, such as ACC (acetyl-CoA carboxylase), and FAS (fatty acid synthase). Since FFA were not oxidized, they favor the re-esterification with glycerol to form triglycerides, VLDL (very low-density lipoprotein) and fat stock intrahepatic, leading to NAFLD [2]. In the present study, we observed an important liver steatosis in all groups fed fructose, fat, or both fructose and fat. These results are consistent with previous reports that indicated that rodents (rats) fed fructose showed large numbers of macro- and micro-intra-hepatic fat vesicles and intralobular inflammation [16].

Our study suggests that an explanation of the increases in intrahepatic triglycerides, accompanied by an increase in the expression of the SREBP-1c protein, and a reduction in the levels of the PPAR-α protein, is responsible for the oxidation of free fatty acids in the liver. Similar results have previously been reported for rats fed a 60% fructose diet for 28 days. These rats demonstrated increased levels of mRNA factors involved in hepatic lipogenesis (ACC, FAS, SREBP-1c and ChREBP) without changes in BM [11]. In addition, in this context, PPAR-γ is a key receptor in glucose homeostasis and lipid metabolism and its overexpression is directly related to the livers with NAFLD [17,18]. Our results show increased expression of both the SREBP-1c and PPAR-γ, and the diminished expression of PPAR-α, which indicate the predominance of the lipogenic pathway against the oxidative pathway in the liver. In the groups HF, HFr and HFHFr we also observed increased expression of proteins involved in hepatic gluconeogenesis, PEPCK, and GLUT2. The literature has demonstrated that the increases in PEPCK suggest hepatic insulin resistance and increased gluconeogenesis, a hallmark of type II diabetes mellitus [19,20]. At normal levels, insulin plays a role in inhibiting G6Pase (glucose 6-phosphatase) and PEPCK. Thus, the overexpression of G6Pase and PEPCK is a response to IR (insulin resistance), as demonstrated in the present study. GLUT2 in the liver is responsible by the influx of glucose in the postprandial period, and the efflux of the substrate in the post-absorptive and fasting periods [21]. In the presence of hyperglycemia, diabetes mellitus type II and NAFLD, the expressions of the gene and the protein GLUT2 mRNA are increased [22,23].

In addition to the lipogenic and oxidative changes in the livers of the fructose-fed groups, we observed the presence of necroinflammatory areas, which are characteristic of NASH. Mice fed high-fat high-carbohydrate (55% fructose and 45% sucrose) have increased hepatic reactive oxygen species and a NASH-like phenotype with significant fibrosis, putting the fructose consumption at the center of necroinflammation and fibrosis in nonalcoholic steatohepatitis [24,25]. This is a concern in public health and leads to an additional effort to diagnose and treat this condition in the population [26,27]. The development of NAFLD is positively correlated with the presence of IR. The IR condition leads to increased serum levels of FFA [28]. Subsequently, the oxidative stress involving lipid peroxidation becomes significant as a precursor of the etiology of NASH [29,30].

The present findings demonstrating the development of NASH in fructose-fed mice have parallels in the recent literature. Mice fed a hypercaloric diet, containing medium-chain trans-fatty acids and fructose, developed liver steatosis with necroinflammatory fibrosis and significant increases in markers of oxidative damage [31]. Moreover, data from mice fed a high-fructose diet, combined or not with high-fat diet, suggest the phenotype of NASH [32].

## 3. Experimental Section

### 3.1. Animal and Diets

The local committee for animal experimentation at the State University of Rio de Janeiro (Rio de Janeiro, Brazil) approved the experimental protocols (CEUA/026/2011).

Male 12-week old C57BL/6 mice were housed in a temperature- (20–23 °C) and humidity-controlled environment with a regular light-dark cycle. The mice had free access to food and water. The procedures were carried out in accordance with Guide for the Care and Use of Laboratory Animals [33], and the Animal Ethics Committee of the State University of Rio de Janeiro (UERJ, Rio de Janeiro, Brazil) approved all of the experimental protocols.

The animals were randomly divided into four dietary groups (*n* = 10 each group). All diets followed the AIN-93M guidelines [34] for rodents for maintenance (Table 2).

Standard chow group (SC, 9% fat, 15% protein, and 76% carbohydrate source: corn starch; 3802.8 kcal/kg), (a) High-fat group [HF, 42% lipids (lipid source: lard), 14% protein, and 44% carbohydrates (carbohydrate source: corn starch); 4702.8 kcal/kg]; (b) High-fructose group (HFr, 9% fat, 15% protein, 76% carbohydrates, 34% fructose; 3802.8 kcal/kg); and (c) High-fat and high-fructose group [HF/HFr, 42% lipids (lipid source: lard), 14% protein and 44% carbohydrates, 34% fructose; 4702.8 kcal/kg].

### 3.2. Body Mass and Food Intake

The intake of food was measured daily (11:00 a.m.), and BM was measured weekly (Monday, 12:00 a.m.). Fresh food was provided daily, and any food remaining from the previous day was discarded.

### 3.3. Oral Glucose Tolerance Test (OGTT) and Intraperitoneal Insulin Tolerance Test (IPITT)

Both the OGTT and IPITT were performed after four months of consumption of each diet. The mice were fasted for six hours (OGTT) or four hours (IPITT) prior to testing. For the OGTT, glucose (1 g/kg) was given orally (orogastric gavage), while for the IPITT, insulin (0.5 units/kg) was given intraperitoneally at time zero. Samples of blood were collected through a small incision at the tail tip at zero, 15, 30, 60 and 120 min for both OGTT and IPITT. The blood glucose concentration was determined with a glucometer (Accu-Chek Active; Roche Applied Science, Sao Paulo, SP, Brazil). The “area under the curve” (AUC, in arbitrary units, a.u.) was calculated to assess glucose intolerance.

### 3.4. Euthanasia

On the day of euthanasia, the animals were fasted for 6 h and were then deeply anesthetized (intraperitoneal sodium pentobarbital, 150 mg/kg). Blood samples were rapidly obtained by cardiac puncture. The liver was dissected, cut and fixed for 48 h in freshly prepared formaldehyde (4% *w/v* in 0.1 M phosphate buffer, pH 7.2) for light microscopy or stored in a −80 °C freezer for Western blot analysis (detailed in Item 3.7).

### 3.5. Plasma Analysis

The plasma was separated from the blood by centrifugation (120 × *g* for 15 min) at room temperature. The plasma total cholesterol (TC), triglycerides (TG), glucose and alanine transaminase (ALT) were determined using a semiautomatic spectrophotometer and respective kits for analysis (Bioclin, Quibasa, Belo Horizonte, MG, Brazil). The plasma insulin was measured using an ELISA kit (Insulin, cat. # EZRMI-13K, Millipore, MO, USA). IR was estimated by the homeostasis model assessment index as [35]:

(1) HOMA-IR=[(insulin×glucose)/22.5]

### 3.6. Liver

Liver fragments were fixed, embedded in Paraplast Plus (Sigma-Aldrich, St. Louis, MO, USA), sectioned at a nominal thickness of 5 μm and stained with hematoxylin and eosin (HE). Ten digital images per animal were analyzed in a random and blinded manner and studied to assess steatosis as previously described [36]. Briefly, we used a 36-point test-system (P_T_) produced by the STEPanizer web-based software (www.stepanizer.com). The volume density (*V*_v_) of liver steatosis was estimated by point-counting the fat droplets in hepatocytes:

(2) $$V_{\text{v}}[\text{steatosis}]=P_{\text{P}}[\text{steatosis}]/{\text{P}}_{\text{T}}$$

where *P*_P_ is the number of points that defined steatosis [37,38]. In addition, the hepatocyte binucleation was measured in a frame of known area.

### 3.7. Immunoblotting

The expression of the sterol regulatory element-binding proteins (SREBP)-1c (Santa Cruz Biotechnology, code sc-367, Santa Cruz, CA, USA), peroxisome proliferator-activated receptors (PPAR)-γ (Santa Cruz Biotecnology, code sc-7273), PPAR-α (Santa Cruz Biotechnology, code sc-9000), glucose transporter (GLUT)-2 (Millipore, cat #07-1402, Billiberica, MA, USA) and phosphoenolpyruvate carboxykinase (PEPCK) (Santa Cruz Biotechnology, code sc-32879), were detected by immunoblotting using rabbit polyclonal antibodies. Approximately 120 mg of liver was homogenized in lysis buffer (pH 6.4) containing protease inhibitors. The protein concentration in the supernatant was determined with a BCA protein assay kit (Thermo Scientific, Rockford, IL, USA). Twenty micrograms of protein were separated by electrophoresis on a 10% polyacrylamide gel (SDS-PAGE) and transferred to a PVDF membrane (GE Healthcare BioSciences, Richmond, CA, USA). The membranes were blocked with 5% non-fat dry milk in Tris-buffered saline (TBS) (Amershan BioSciences, Uppsala, Sweden) containing 0.05% Tween-20 (Bio Rad, Hercules, CA, USA) and then incubated overnight at 4 °C with anti-SREBP-1c, anti-PPAR-γ, anti-PPAR-α, anti-GLUT2 and anti-PEPCK. Subsequently, the membranes were washed three times with TBS containing 0.05% Tween-20 and incubated with secondary antibody for one hour. Structural protein β-actin (Santa Cruz Biotechnology, code sc-81178) was obtained by stripping the PVDF membrane proteins of the liver and was used to correct the expressions of the proteins mentioned. The bands were detected by chemiluminescence using the ECL reagent kit (GE Healthcare BioSciences) and obtained by ChemiDoc imaging system (Bio Rad). The density of the signals was analyzed using ImageJ software version 1.47q (National Institutes of Health, NIH, Bethesda, MD, USA).

### 3.8. Data Analysis

The data are expressed as the means and the respective standard errors of the mean. All samples were tested for normality and homogeneity of the variances, and the differences among the groups were then tested with a one-way ANOVA and the *post-hoc* test of Holm-Sidak (GraphPad Prism version 6.03 for Windows, San Diego, CA, USA). A *p*-value of ≤0.05 was considered statistically significant.

## 4. Conclusions

Mice fed a high-fat diet, a high-fructose diet or a combination of high-fat and high-fructose, develop various components of the human metabolic syndrome. The metabolic responses to the consumption of a diet high in fat are already widely discussed in the literature. This study is relevant in demonstrating that fructose consumption, even in the absence of obesity, causes serious and deleterious changes in the liver with the presence of the dyslipidemia, IR and NAFLD with areas of necroinflammation. These conditions are associated with a poor prognosis.

## Figures and Tables

**Table 1:** Food intake and plasma biochemical responses. Values are means ± standard error. Symbols represent significant differences from ^a^ SC, *p* ≤ 0.05; ^b^ HF, *p* ≤ 0.05; and ^c^ HFr, *p* ≤ 0.05, assessed using one-way ANOVA with *post-hoc* Holm-Sidak testing. Groups: SC (standard chow); HF (high-fat diet); HFr (high-fructose diet); HF/HFr (high-fat-high-fructose diet).

Data	Groups			

	SC	HF	HFr	HF/HFr
Alanine aminotransferase (mg/dL)	35.05 ± 7.81	81.93 ± 6.53 ^a^	75.20 ± 5.66 ^a^	70.97 ± 4.92 ^a^
Energy intake (kcal/animal/week)	12330 ± 204	13741 ± 348 ^a^	12126 ± 427 ^b^	13565 ± 216 ^a^,^c^
Food Intake (g/animal/week)	3.24 ± 0.40	2.92 ± 0.37	3.18 ± 0.56	2.88 ± 0.23
Glucose (mmol/L)	5.08 ± 0.70	12.31 ± 1.14 ^a^	9.98 ±0.88 ^a^	10.20 ± 1.04 ^a^
Hepatic TG (mg/dL)	74.8 ± 1.4	126.2 ± 4.6 ^a^	93.0 ± 1.8 ^a^,^b^	110.9 ± 6.1 ^a^,^b^,^c^
HOMA-IR	1.0 ± 0.14	4.1 ± 0.35 ^a^	3.1 ± 0.32 ^a^	3.1 ± 0.31 ^a^
Insulin (μIU/mL)	4.6 ± 0.11	7.8 ± 0.50 ^a^	6.9 ± 0.36 ^a^	6.8 ± 0.49 ^a^
IPITT (AUC, a.u.)	601.7 ± 59.9	817.0 ± 23.4 ^a^	896.9 ± 22.3 ^a^	829.8 ± 73.0 ^a^
OGTT (AUC, a.u.)	1086.2 ± 21.2	1249.2 ± 24.5 ^a^	1261.8 ± 42.8 ^a^	1214.8 ± 32.5 ^b^,^c^
TC (mg/dL)	74.2 ± 5.6	110.8 ± 5.9 ^a^	101.8 ± 3.8 ^a^	124.5 ± 8.4 ^a^
TG (mg/dL)	58.0 ± 1.4	66.0 ± 0.5 ^a^	66.8 ± 0.9 ^a^	66.4 ± 0.7 ^a^

Abbreviations: a.u. (arbitrary units); AUC (area under the curve); HOMA-IR (homeostasis model assessment for insulin resistance); IPITT (intraperitoneal insulin tolerance test); OGTT (oral glucose tolerance test); TC (total cholesterol); TG (triglycerides).

**Table 2:** Diet Composition. Groups: SC, HF, HFr, HF/HFr.

Nutrient (g/Kg)	Diets			

	SC	HF	HFr	HF/HFr
Casein (≥85% protein)	140.0	160.0	140.0	160.0
Cornstarch	620.7	420.69	296.8	20.7
Sucrose	100.0	100.0	100.0	100.0
Fructose	–	–	323.85	400.0
Soybean Oil	40.0	40.0	40.0	40.0
Lard	–	180.0	–	180.0
Fiber	50.0	50.0	50.0	50.0
Vitamin Mix	10.0	10.0	10.0	10.0
Mineral Mix	35.0	35.0	35.0	35.0
Cystine	1.8	1.8	1.8	1.8
Coline	2.5	2.5	2.5	2.5
Antioxidant	0.008	0.008	0.008	0.008
Total (g)	1.000	1.000	1.000	1.000
Energy (kcal/Kg)	3802.8	4702.8	3802.8	4702.8
Carbohydrate (%)	76	44	76	44
Fructose (%)	–	–	34	34
Protein (%)	15	14	15	14
Lipid (%)	9	42	9	42
